# Cardioprotective Properties of Human Platelets Are Lost in Uncontrolled Diabetes Mellitus: A Study in Isolated Rat Hearts

**DOI:** 10.3389/fphys.2018.00875

**Published:** 2018-07-10

**Authors:** Isabella Russo, Saveria Femminò, Cristina Barale, Francesca Tullio, Stefano Geuna, Franco Cavalot, Pasquale Pagliaro, Claudia Penna

**Affiliations:** ^1^Department of Clinical and Biological Sciences, AOU San Luigi, University of Turin, Turin, Italy; ^2^Istituto Nazionale Ricerche Cardiovascolari (INRC), Bologna, Italy; ^3^Internal Medicine and Metabolic Disease Unit, AOU San Luigi, University of Turin, Turin, Italy; ^4^Ospedale San Luigi Gonzaga, Orbassano, Italy

**Keywords:** cardioprotection, infarct size, ischemia/reperfusion, platelets, sphingosine-1-phosphate, type 2 diabetes mellitus

## Abstract

Platelets affect myocardial damage from ischemia/reperfusion. Redox-dependent sphingosine-1-phosphate production and release are altered in diabetic platelets. Sphingosine-1-phosphate is a double-edged sword for ischemia/reperfusion injury. Therefore, we aimed to verify whether: (1) human healthy- or diabetic-platelets are cardioprotective, (2) sphingosine-1-phosphate receptors and downstream kinases play a role in platelet-induced cardioprotection, and (3) a correlation between platelet redox status and myocardial ischemia/reperfusion injury exists. Isolated rat hearts were subjected to 30-min ischemia and 1-h reperfusion. Infarct size was studied in hearts pretreated with healthy- or diabetic-platelets. Healthy-platelets were co-infused with sphingosine-1-phosphate receptor blocker, ERK-1/2 inhibitor, PI3K antagonist or PKC inhibitor to ascertain the cardioprotective mechanisms. In platelets we assessed (i) aggregation response to ADP, collagen, and arachidonic-acid, (ii) cyclooxygenase-1 levels, and (iii) AKT and ERK-phosphorylation. Platelet sphingosine-1-phosphate production and platelet levels of reactive oxygen species (ROS) were quantified and correlated to infarct size. Infarct size was reduced by about 22% in healthy-platelets pretreated hearts only. This cardioprotective effect was abrogated by either sphingosine-1-phosphate receptors or ERK/PI3K/PKC pathway blockade. Cyclooxygenase-1 levels and aggregation indices were higher in diabetic-platelets than healthy-platelets. Diabetic-platelets released less sphingosine-1-phosphate than healthy-platelets when mechanical or chemically stimulated *in vitro*. Yet, ROS levels were higher in diabetic-platelets and correlated with infarct size. Cardioprotective effects of healthy-platelet depend on the platelet’s capacity to activate cardiac sphingosine-1-phosphate receptors and ERK/PI3K/PKC pathways. However, diabetic-platelets release less S1P and lose cardioprotective effects. Platelet ROS levels correlate with infarct size. Whether these redox alterations are responsible for sphingosine-1-phosphate dysfunction in diabetic-platelets remains to be ascertained.

## Introduction

The role played by platelets in determining cardiac ischemia/reperfusion (I/R) injury is not clear. Studies, conducted in the nineties, suggested that healthy platelets have cardioprotective properties. Indeed, healthy rat platelets resulted protective against myocardial injury induced by I/R in isolated rat hearts and by hypoxia/reoxygenation in cultured adult rat cardiomyocytes (Yang et al., 1993, 1994, 1998, 1999; Yang and Mehta, 1994; Mehta et al., 1999). Subsequently, in a 2002 study, Mirabet et al. (2002) infused pig platelets – collected before or after coronary occlusion – into Langendorff rat hearts, which were subsequently submitted to I/R. Only the pig platelets collected after reperfusion enhanced I/R injury in rat hearts. The authors concluded that the deleterious effect of platelets on reperfused myocardium depends on their activation status.

Recent studies by Cohen and Downey group have shown that P2Y_12_ receptor antagonists (e.g., Cangrelor, Ticagrelor) targeting platelets may exert a cardioprotective effect. Since the cardioprotective mechanism seems not attributable to inhibition of platelet aggregation (Yang et al., 2013a,b; Cohen and Downey, 2014, 2015), the authors suggest that patients treated with these antagonists are benefiting from protective “conditioning pathways” triggered by factors released by platelets. Indeed, these conditioning pathways are very similar to those activated by brief periods of intermittent non-lethal ischemia, before or after a prolonged ischemia, that are the well-known phenomena of pre- and post-conditioning (Cohen and Downey, 2015; Pagliaro and Penna, 2015; Penna et al., 2015). These conditioning phenomena induce the activation of protective pathways and a consequent reduction of I/R injury.

Many endogenous *molecules* could induce conditioning cardioprotection, eliciting specific pathways (Cohen and Downey, 2015; Pagliaro and Penna, 2015; Penna et al., 2015). Platelets are known to release a plethora of molecules, which mediate platelet effects in a variety of conditions, including cardioprotection against I/R injury (Yang et al., 1993; Penna et al., 2005, 2015). A platelet-derived cardioprotective molecule is *Sphingosine 1-phosphate* (S1P), a bioactive lipid mediator abundantly carried and stored by platelets, whose production and release seem *redox-dependent* and related to thromboxane formation, but mechanisms are poorly understood (Yatomi et al., 1995; Tani et al., 2005; Ulrych et al., 2011; Ono et al., 2013; Russo et al., 2017).

S1P exerts its function through five specific cell surface receptors (S1PRs) on target organs (Tani et al., 2005; Vito et al., 2016) and is a double-edged sword: S1P is a potent proinflammatory and mediator of allergic diseases (Maceyka and Spiegel, 2014; Kulinski et al., 2016; Vito et al., 2016), but it is also a potent protectant against I/R injury (Vessey et al., 2009). The S1P cardioprotection seems mediated by interaction with types 1–3 S1PRs, with subsequent activation of the so-called RISK/SAFE pathways (Vessey et al., 2009; Cohen et al., 2016). However, S1PR_1_ does not appear cardioprotective *in vivo*, whereas a combination of S1PR_2_ and S1PR_3_ activation mediates cardioprotection (Means et al., 2007). Nevertheless, these studies revealed that S1P is an important mediator of cardioprotection and could induce either pre- or post-conditioning.

Several pathological conditions, including type 2 diabetes mellitus (T2DM), reduce the efficacy of ischemic conditioning strategies (Ferdinandy et al., 2014). However, unlike cardioprotection induced by ischemic conditioning, P2Y_12_ antagonist-induced conditioning can result in significant attenuation of I/R injury also in diabetic animals (Bell et al., 2015). These last results open an interesting scenario on the role of platelets in cardioprotection. Therefore, in-depth studies on platelet characteristics and their role in cardioprotection are necessary.

Of note, the S1P dynamic is profoundly altered in diabetic conditions (Fox et al., 2011) and diabetes is indicated as a “pro-thrombotic state” in which, among other alterations, *oxidative stress* and *platelet hyperactivity* play crucial roles in the cardiovascular complications (Jung et al., 2015). Therefore, one can wonder: are diabetic platelets cardioprotective and can reduce I/R damage? Although it is well known that diabetic platelets display a certain degree of activation (Watala et al., 2005; Jung et al., 2015), to the best of our knowledge, no studies ascertained whether the platelet-dependent cardioprotective properties are altered in the platelets of T2DM subjects.

Therefore, after a thorough study of platelet characteristics derived from healthy and T2DM subjects, we aimed to verify whether:

(1) *Human* healthy and diabetic platelets are cardioprotective in an isolated rat heart model;
(2) S1P and RISK-pathways play a role in platelet-induced cardioprotection;
(3) a correlation between platelet redox status (an index of platelet dysfunction) and myocardial I/R injury exists.

To these aims, blood samples were collected from voluntary healthy subjects and from T2DM patients. Platelet samples were analyzed for aggregatory aspects, ROS production and S1P release *in vitro* or were used to be infused in isolated hearts before being subjected to I/R protocols either in the absence or in the presence of S1P receptor blocker and RISK-pathway antagonists.

## Materials and Methods

### Subjects and Platelet Preparation

The study was performed on platelets obtained from 35 healthy volunteers and 16 type 2 diabetes mellitus (T2DM) patients. Healthy subjects were not on any antiplatelet therapy nor did they have any history of cardiovascular disease, metabolic syndrome or diabetes. Patients affected by T2DM were recruited among those attending the Metabolic Disease and Diabetes Unit of the San Luigi Gonzaga Hospital in Orbassano (Turin); six of them were at first diagnosis, the others were poorly controlled with glycated hemoglobin A1c (HbA1c) > 7.5% even though on antidiabetic drugs. All subjects were without previous cardiovascular events and antiplatelet drugs in the previous 2 weeks. The totality of subjects gave informed consent before the investigation and the Ethics Committee of San Luigi Hospital approved the study, in accordance with the Declaration of Helsinki. The present study is a “case-control study” that raises potential issues of bias due to patient- and voluntary-donor selection. However, data collection was performed by blinded operators on the origin of platelets. Therefore, there is no subjectivity implied in the assessment or bias under these conditions.

Blood samples were collected from the antecubital vein and laboratory measurements were performed after a 12-h overnight fast. Biochemical parameters, including assessment of fasting glucose, total cholesterol, triglycerides, low-density lipoprotein (LDL) cholesterol, high-density lipoprotein (HDL) cholesterol and HbA1c as well as platelet number were measured using routine laboratory methods, performed by the central laboratory of our Hospital.

For studies concerning platelet function, a venous blood sample was withdrawn without stasis after an overnight fast, and anticoagulated with 3.8% sodium citrate, pH 7.4 (vol/vol: 1/9) for aggregation studies in platelet-rich plasma or with a citrate-dextrose solution (ACD; v/v, 1/6) for experiments on washed platelets. Platelet-rich plasma was obtained by using Platelet Function Centrifuge (BioData Corporation, Horsham, PA, United States) designed to provide a rapid separation of platelet-rich plasma by a centrifugation for 30 s and of platelet-poor plasma by a further centrifugation for 120 s. To prepare washed platelets, ACD-anticoagulated platelet-rich plasma, obtained by centrifugation at 100 × *g* for 20 min, underwent further centrifugation at 2000 × *g* for 10 min and pellet was washed two times at 37°C in HEPES-Na buffer (in mmol/L) (10 HEPES Na, 140 NaCl, 2.1 MgSO_4_, 10 D-glucose, pH 7.4).

### Platelet Aggregation Studies

Platelet aggregation studies were carried out in both platelet-rich plasma and whole blood. Aggregation tests in platelet-rich plasma followed light-scattering changes as described by Born (1962) using an eight-channel aggregation system (Platelet Aggregation Profiler, Model PAP-8, BioData Corporation) and were induced by ADP (10 μmol/L), arachidonic acid (AA, 1 mmol/L), and collagen (4 mg/L). Each aggregation test was recorded for 5 min and reported as percent of maximal aggregation.

For platelet aggregation in whole blood by impedance method, citrated blood samples were diluted 1:1 with physiologic saline and the tests were performed in a Chrono-Log Whole Blood Aggregometer, Model 500 at a constant stirring of 1000 rpm. Aggregation was induced with ADP (20 μmol/L), collagen (4 mg/L) or AA (1 mmol/L) and was recorded for 8 min. The maximum increase in resistance, expressed in ohms, was calculated.

#### Reactive Oxygen Species (ROS) Assay

Intracellular ROS were evaluated in washed platelets by using the sensitive fluorescent indicator DCF-DA oxidized by H_2_O_2_ to the highly fluorescent DCF (Eruslanov and Kusmartsev, 2010). Washed platelets (6 × 10^7^ ml^-1^) were exposed to 10 μmol/L cell permeant reagent DCF-DA and DCF fluorescence was measured at basal and after stimulation with AA (100 μmol/L) added just before measuring fluorescence. To examine specific signaling in basal ROS generation, platelets were preincubated for 20 min with the NADPH-oxidase inhibitors APO (10 μmol/L) or DPI (10 μmol/L), or the cyclooxygenase-1 (COX-1) inhibitor indomethacin (INDO, 100 μmol/l) (Gerrits et al., 2010; Barale et al., 2017).

Fluorescence was measured over a 60-min period at 1-min intervals using a plate fluorometer (GloMax-Multi Detection System, Promega Corporation, Madison, WI, United States) fitted with 490 nm excitation and 520 nm emission filters. Fluorescence per minute was calculated for each sample.

#### Platelet S1P Assay

Washed platelets (500 μl for each sample containing 150 × 10^6^ platelets) were used to measure S1P release both in the absence (resting platelets) and in the presence of a stir bar put into the cuvette (stirring platelets) (Lee et al., 2016). For samples subjected to stirring, the stir bar inside the sample is spun, thus stirring the washed platelets to a 1200 rpm speed at 37°C. An eight-channel optical aggregometer was used (Platelet Aggregation Profiler, Model PAP-8, BioData Corporation) at this end. After 8-min incubation with or without collagen (4 mg/l), all samples were submitted to repeated freeze-thaw cycles to let out the inside components, centrifuged at 3000 rpm for 20 min and supernatants were collected for S1P measurement.

S1P levels were determined by using an Enzyme-Linked Immuno-adsorbent Assay according to manufacturer instructions (Bioassay Technology Laboratory, Shanghai, China). Standard curve range was 5–1500 ng/l, the sensitivity of the kit was 2.34 ng/l, and intra- and inter-assay CV were <8% and <10%, respectively.

#### Western Blot Analysis in Platelets

To assess the activation state of platelets, we detected the phosphorylation/activation of phosphatidylinositol-3-kinases (PI3K) and extracellular signal-regulated kinases (ERK) in response to platelet agonists: washed platelets were stimulated by collagen (4 mg/L) or AA (100 μmol/L) for 8 min and the amounts of AKT, and pAKT, and ERK-2, pERK-1/2 were evaluated, respectively.

Cyclooxygenase-1 expression was also determined. Washed platelets samples were centrifuged and pellets solubilized in Laemmli buffer and processed as previously described (Russo et al., 2007). Membranes were incubated with rabbit anti-AKT, mouse anti-ERK-2 (Cell Signaling, Danvers, MA, United States), rabbit anti-phospho-Akt-Ser-473, mouse anti-phospho-ERK-1/2-Tyr-204, or mouse anti-COX-1 (Santa Cruz Biotechnology). As secondary antibodies, we used goat anti-mouse (Jackson Immuno Research Lab., West Grove, PA, United States) or goat anti-rabbit (Cell Signaling) antibodies conjugated to horseradish peroxidase. Blots were scanned and densitometrically analyzed by the image analyzer 1D Image Analysis software (Kodak, Rochester, NY, United States).

### Animals

Male Wistar rats (*n* = 75; 4–5-month-old, body weight 400–450 g, Harlan Laboratories Udine, Italy) were used for the *ex vivo* experiments as specified below. Animals were housed under controlled conditions with free access to standard rat diet and tap water. Rats received humane care in compliance with the European Directive 2010/63/EU on the protection of animals used for scientific purposes. The animal protocols followed in this study were approved by the local “Animal Use and Care Committee.” In this study, six hearts were discarded due to the very low left ventricular developed pressure or other technical issues after connection to the perfusion line.

#### Isolated Heart Preparations

Rats were anesthetized, heparinized (800 U/100 g b.w., i.m.), and hearts were rapidly excised, placed in an ice-cold buffer solution, and weighed. Isolated hearts were attached to the perfusion apparatus and retrogradely perfused with oxygenated Krebs–Henseleit buffer solution (KHS) containing (in mM): 127 NaCl, 5.1 KCl, 17.7 NaHCO_3_, 1.26 MgCl_4_, 1.5 CaCl_2_, 11 D-glucose (pH 7.4; 37°C; 95% O_2_/5% CO_2_). Hearts were kept in a temperature-controlled chamber (37°C), electrically paced at 280 b.p.m. The hearts were perfused in constant-flow mode and to assess the preparation conditions, coronary perfusion pressure and left ventricular pressure were monitored during all experiments (Penna et al., 2005, 2012).

#### Experimental Groups

**FIGURE 1:**
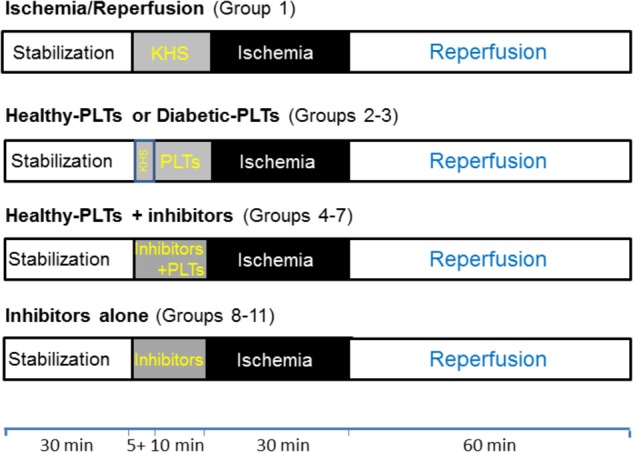
Timeline of experimental protocols. Protocols of ischemia (black boxes) and reperfusion (white boxes) in isolated rat hearts. After stabilization (white boxes), Krebs-Henselite solution (KHS) was infused *via* a collateral line alone or with addition of washed PLTs and/or inhibitors (gray boxes). Washed platelets (PLTs) were given for 10 min before ischemia and inhibitors were given 5 min before PLTs and were stopped at the end of the 10 min of infusion of PLTs (total time infusion for inhibitors was 15 min). For further explanations, see the text.

To verify the cardioprotective properties of washed PLTs derived from healthy subjects (healthy-PLTs) or diabetic patients (diabetic-PLTs), hearts were assigned to one of the experimental groups described below on the basis of availability of the platelet donor. In all groups, the hearts were subject to 30 min stabilization, and 30 min of normothermic global ischemia followed by 60 min reperfusion (I/R). Protocols are as follow (**Figure 1**):

(1) In the I/R group (*n* = 7), after stabilization, only the I/R protocol was performed (Bell et al., 2011);
(2) In the healthy-PLT group (*n* = 8) healthy subject washed platelets diluted in KHS (healthy-PLTs, 3 × 10^7^ ml^-1^) were infused to the heart, via a collateral line, for 10 min at a flow rate of 12 ml/h; then hearts were subjected to I/R protocol;
(3) In the diabetic-PLT group (*n* = 8) diabetic patient washed platelets diluted in KHS (diabetic-PLTs, 3 × 10^7^ ml^-1^) were infused to the heart, via a collateral line, for 10 min at a flow rate of 12 ml/h; then hearts were subjected to I/R protocol.

For groups 2 and 3, each rat heart was treated with the platelets from a single donor and the platelet concentration and time of infusion were similar to those used by Yang et al. (1993). This relatively low concentration of platelets reduces the risk of capillary plugging and loss of myocardial function.

Since only healthy-PLTs affected significantly infarct size (see section “Results”), the following healthy-PLT+inhibitors groups were studied. In these groups, hearts were subjected to infusion of inhibitor alone for 5 min, then a co-infusion with healthy-PLT, for 10 min as in group 2, was performed, and, finally, the hearts underwent I/R protocol. Therefore, inhibitors were infused for a total of 15 min:

(4) In the healthy-PLT+VPC group (*n* = 5), to ascertain the involvement of S1P receptors in healthy-PLT-induced cardioprotective mechanisms, we used the S1P receptor blocker VPC23019 (1 × 10^-6^ M) as S1P1/3 receptor antagonist (Davis et al., 2005; Vessey et al., 2009);
(5) In the healthy-PLT+U0126 group (*n* = 5), to ascertain the involvement of myocardial ERK1/2, we used the ERK1/2 antagonist, U0126 (60 × 10^-6^ M) (Mochizuki et al., 2013);
(6) In the healthy-PLT+LY group (*n* = 5), to ascertain the involvement of myocardial PI3K/AKT, we used the PI3K antagonist, LY294002 (5 × 10^-5^ M) (Penna et al., 2005);
(7) in the healthy-PLT+CHE group (*n* = 5), to ascertain the involvement of myocardial PKC, we used the PKC antagonist, Chelerythrine (1 × 10^-6^ M) (Penna et al., 2017);

Inhibitors were also tested alone at the same concentrations and for the same periods:

(8) In the VPC23019 group (*n* = 5): hearts were subjected to infusion of VPC23019 for 15 min; then hearts were subjected to I/R protocol;
(9) In the U0126 group (*n* = 4): hearts were subjected to infusion of U0126 for 15 min; then were subjected to I/R protocol;
(10) In the LY294002 group (*n* = 4): hearts were subjected to infusion of LY294002 for 15 min; then hearts were subjected to I/R protocol;
(11) In the Chelerythrine group (*n* = 4): hearts were subjected to infusion of Chelerythrine for 15 min; then hearts were subjected to I/R protocol.

#### Infarct Size Assessment

In order to assess infarct size extension, a gravimetric method was used in a blinded fashion. In briefly at the end of reperfusion, hearts were rapidly removed from perfusion apparatus and the left ventricular tissue was dissected into 2–3 mm circumferential slices. After 20 min of incubation at 37°C in 0.1% solution of nitro-blue tetrazolium in phosphate buffer, unstained necrotic tissue was carefully separated from stained viable tissue and weighed. The necrotic mass was expressed as a percentage of total left ventricular mass, which was considered as risk area (Penna et al., 2014, 2017).

#### Western Blot Analysis in Hearts

In additional experiments, immunoblot procedures and analyses were conducted as previously described (Penna et al., 2012, 2014, 2017). Immediately after the hearts had undergone I/R (*n* = 4) or PLT pretreatment (either healthy-PLTs, *n* = 8, or diabetic-PLTs *n* = 5), the cardiac specimens were frozen in liquid nitrogen before being stored at -80°C until protein extraction. Myocardial tissues of the above groups were homogenized in a frozen RIPA lysis buffer (Sigma-Aldrich, St. Louis, MO, United States) containing a mixture of protease inhibitors (1 mM of aprotinin, 20 mM of phenylmethylsulfonyl fluoride and 200 mM of sodium orthovanadate). Subsequently, myocardial homogenates were centrifuged at 15,000 × *g* for 25 min at 4°C for debris removal. Protein concentration was assessed using a Bradford reagent following the procedure described by the manufacturer (Sigma-Aldrich, St. Louis, MO, United States). Equivalent amounts of proteins (80 μg) were loaded and electrophoresed on SDS polyacrylamide gel and transferred to a polyvinylidene difluoride membrane. Membranes were then incubated overnight at 4°C with primary antibodies mouse anti-phospho-ERK-1/2-Tyr-204 (Santa Cruz Biotechnology) or mouse anti-ERK-1/2 (Cell Signaling, Danvers, MA, United States). As secondary antibody, we used goat anti-mouse (Jackson Immuno Research Lab., West Grove, PA, United States). Blots were scanned and densitometrically analyzed by the image analyzer ID Image Analysis software (Kodak, Rochester, NY, United States).

### Chemicals

Collagen and arachidonic acid were purchased from Mascia Brunelli Spa (Monza, Milan, Italy). The sources of the specific antibodies are shown in the different sections. The other reagents were obtained from Sigma (St. Louis, MO, United States) if not differently specified.

### Statistical Analysis

Data are expressed as mean ± SE. Statistical analysis was performed with one-way analysis of variance (ANOVA) followed by Student’s *t*-test or Newman–Keuls multiple-range test depending on the experiments. *P*-value ≤ 0.05 was considered to be significant. A linear fit is assessed between ROS production and infarct size. A *P*-value < 0.05 was considered statistically significant.

## Results

The characteristics of study participants are shown in **Table 1**. Healthy volunteers and T2DM subjects were significantly different for BMI (*p* < 0.0001), HbA1c (*p* < 0.0001), fasting glucose (*p* < 0.0001), total cholesterol (*p* < 0.0001), triglycerides (*p* < 0.005), and LDL cholesterol (*p* < 0.002). There were no differences between the two groups with regard to age, HDL-cholesterol, platelet number, systolic and diastolic blood pressure.

**Table 1:** Clinical characteristics, laboratory findings, and medications of healthy and T2DM subjects.

	Healthy subjects (*n* = 35)	T2DM subjects (*n* = 16)	*p*-value
M/F	17/18	7/9	
Age (years)	53.0 ± 1	57.1 ± 2	0.08
BMI (kg/m^2^)	23.6 ± 0.2	31.6 ± 2	0.0001
HbAlc (%)	5.4 ± 0.1	11.3 ± 0.8	0.0001
Fasting glucose (mg/dl)	84.9 ± 2	320.5 ± 36	0.0001
Total cholesterol (mg/dl)	155.2 ± 4	193.9 ± 13	0.0001
HDL cholesterol (mg/dl)	52.0 ± 1	48.2 ± 2	0.074
Triglycerides (mg/dl)	105.0 ± 4	151.9 ± 22	0.005
LDL cholesterol (mg/dl)	85.7 ± 4	115.4 ± 10	0.002
SBP (mmHg)	120.6 ± 1	122.1 ± 8	0.786
DBP (mmHg)	78.5 ± 0.8	75.6 ± 2	0.093
Platelets (×l0^3^/μl)	236.2 ± 5	243.9 ± 8	0.401
Insulin use	–	6 (38%)	
Oral antidiabetic medication use	–	9 (56%)	
Statin use	–	10 (63%)	
Antihypertensive drugs use	–	11 (69%)	

*HDL, high-density lipoprotein; LDL, low-density lipoprotein; BMI, body mass index; SBP, systolic blood pressure; DBP, diastolic blood pressure.*

### Platelet Characteristics

We first analyzed platelet (PLT) characteristics in both T2DM and healthy subjects.

#### Platelet Aggregation Parameters Were Higher in Diabetic-PLTs Than in Healthy-PLTs

**FIGURE 2:**
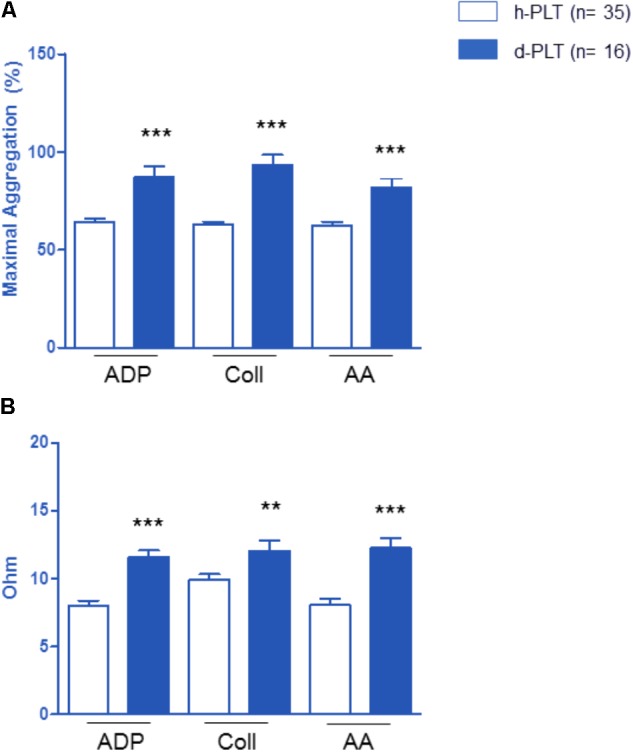
Effect of platelet exposure to different pro-aggregating agents: ADP, collagen (Coll) and arachidonic acid (AA). Platelet aggregation parameters were higher in platelets of diabetic patients (d-PLT) than in those of healthy subjects (h-PLT), in both platelet-rich plasma **(A)** or washed platelets **(B)**. ^∗∗^*p* < 0.005, ^∗∗∗^*p* < 0.0001 vs. h-PLT (unpaired *t*-test).

Platelet aggregation parameters were studied in diabetic-PLTs and in healthy-PLTs, in both platelet-rich plasma (**Figure 2A**) and whole blood (**Figure 2B**) samples. As shown in **Figure 2A**, in platelet-rich plasma, diabetic-PLTs, compared to healthy-PLTs, showed significantly higher aggregation to ADP (*p* < 0.0001), collagen (*p* < 0.0001), and AA (*p* < 0.0001). In particular, in diabetic-PLTs, the aggregation was higher by about 35% in response to ADP, 47% to collagen, and 30% to AA.

As shown in **Figure 2B**, in whole blood, diabetic-PLTs, compared to healthy-PLTs, showed significantly higher aggregation to ADP (*p* < 0.0001), to collagen (*p* < 0.005), and AA (*p* < 0.0001). Specifically, in diabetic-PLTs, the aggregation was higher by about 45% in response to ADP, 21% to collagen, and 53% to AA.

#### S1P Release Was Lower in Diabetic-PLTs Than in Healthy-PLTs

**FIGURE 3:**
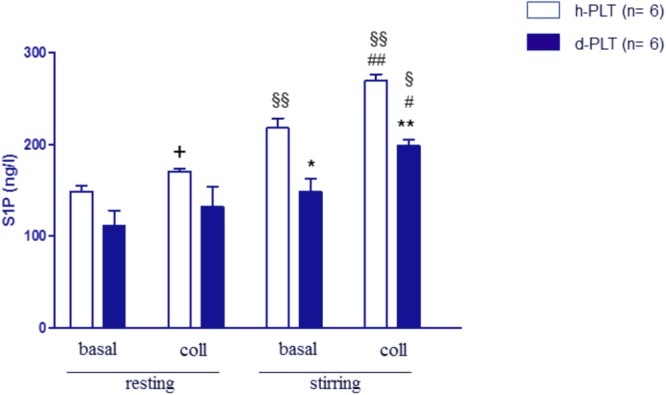
S1P release in washed platelets, in resting and stirring conditions. In resting conditions, S1P levels were not different between platelets from T2DM patients (d-PLT) and platelets from healthy subjects (h-PLT). +*p* < 0.05 vs. resting basal. In stirring samples, S1P levels were lower in d-PLT than in h-PLT both in the absence and in the presence of collagen. ^∗^*p* < 0.001, ^∗∗^*p* < 0.0001 vs. healthy-PLT (unpaired *t*-test); ^#^*p* < 0.01, ^##^*p* < 0.005 vs. corresponding stirring basal; ^§^ *p* < 0.005, ^§§^ *p* < 0.001 vs. corresponding resting conditions (Newman–Keuls multiple-range test).

The release of S1P was measured in supernatants of washed platelets in *resting* and *stirring* conditions (**Figure 3**). In *resting* platelet samples, S1P levels did not differ significantly between healthy- and diabetic-PLTs both in the absence (149 ± 6 vs. 112 ± 16 ng/l, *p* = ns) and in the presence of collagen (171 ± 3 vs. 132 ± 21 ng/l, *p* = ns).

When samples were subjected to *stirring*, a significant increase in S1P levels was observed in healthy-PLTs (from 149 ± 6 to 218 ± 10 ng/l, *p* < 0.0001) but not in diabetic-PLTs (from 112 ± 16 to 148 ± 13 ng/l, *p* = ns). Furthermore, S1P levels, in stirring samples, were lower in diabetic-PLTs than in healthy-ones both in the absence (148 ± 13 vs. 218 ± 10 ng/l, *p* < 0.001) and in the presence of collagen (199 ± 6 vs. 270 ± 7 ng/l, *p* < 0.0001).

#### ROS Levels Were Higher in Diabetic-PLTs Than in Healthy-PLTs

**FIGURE 4:**
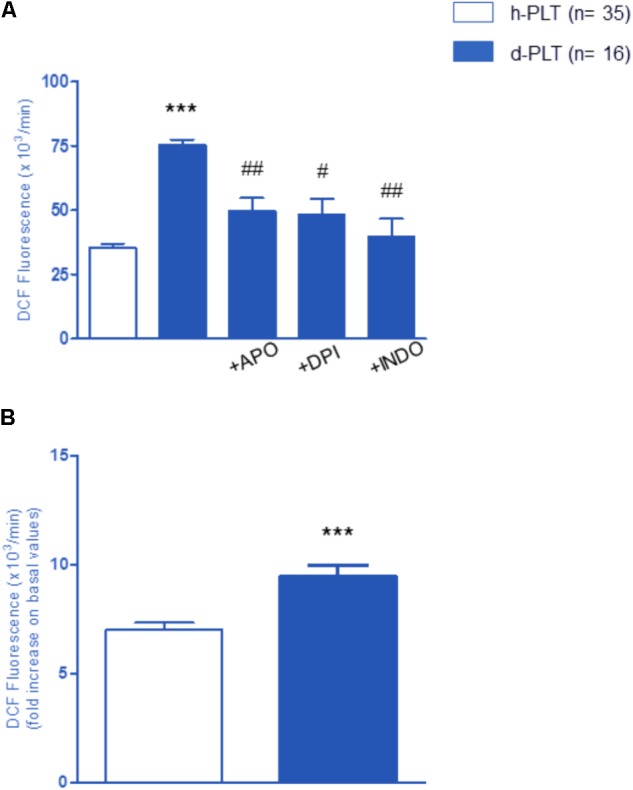
Variation of the intra-platelet level of ROS measured by DCF fluorescence. **(A)** ROS level in resting platelets of diabetic patients (d-PLT) were higher than in resting platelets of healthy subjects (h-PLT). 20-min preincubation with apocynin (APO), diphenyleneiodonium (DPI), or indomethacin (INDO) reduced the levels of ROS produced by d-PLT. **(B)** PLT stimulation with arachidonic acid increased ROS levels in both h-PLTs and d-PLTs. ^∗∗∗^*p* < 0.0001 vs. h-PLT; ^#^*p* < 0.005, ^##^*p* < 0.001 vs. resting d-PLT (paired *t*-test).

To ascertain whether higher aggregation parameters were associated with higher ROS levels, we used 2′,7′-dihydrodichlorofluoresceine diacetate (DCF-DA) oxidation to detect ROS intracellular concentration. As shown in **Figure 4A**, there was a significantly higher ROS level in resting, non-stimulated diabetic-PLTs than in controls (*p* < 0.0001).

To identify the origin of ROS, measurements were repeated after a 20-min pre-incubation with either apocynin (APO) or diphenyleneiodonium (DPI), two different NADPH-oxidase inhibitors, as well as the COX-1 inhibitor, Indomethacin (INDO). Data revealed that the basal ROS production in diabetic-PLTs is, at least in part, mediated by the activity of NADPH-oxidase and COX-1. In fact, with regard to baseline values, we observed a significant decrease of ROS production in the presence of NADPH-oxidase inhibitors, APO (*p* < 0.001) or DPI (*p* < 0.005), and COX-1 antagonist, INDO (*p* < 0.001). As expected, platelet stimulation with AA increased ROS levels in both healthy-PLTs and diabetic-PLTs (**Figure 4B**). Furthermore, diabetic-PLTs generated a significantly higher ROS amount after stimulation with AA (6 vs. 9 fold increase, *p* < 0.0001).

#### COX-1 Expression and Signaling Transduction Molecules Were Up-Regulated in Diabetic-PLTs

**FIGURE 5:**
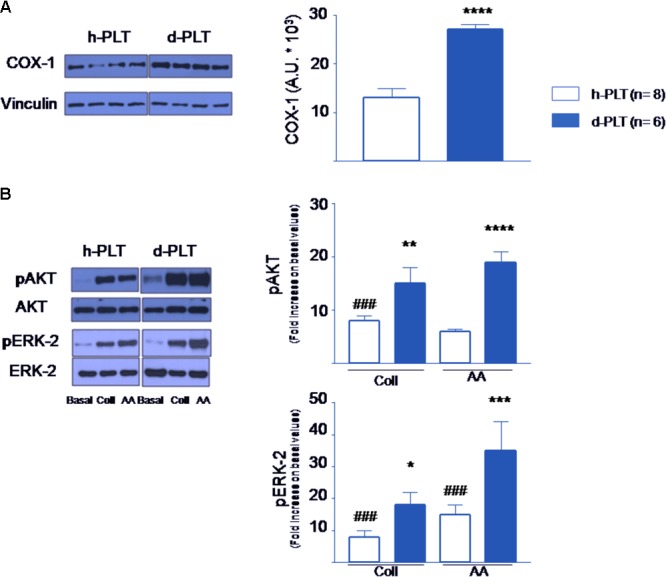
Protein content in platelets determined by Western blot. **(A)** Cyclooxygenase-1 (COX-1) levels were higher in platelets of diabetic patients (d-PLT) than in those of healthy subjects (h-PLT). **(B)** Phosphorylated Protein Kinase B (pAKT) and Phosphorylated Extracellular Signal-regulated Kinase-2 (pERK-2) were increased by collagen (Coll) and arachidonic acid (AA) stimulation in both h-PLT and d-PLT. However, the increases were higher in d-PLTs than in h-PLTs. ^###^*p* < 0.0001 vs. basal level; ^∗^*p* < 0.05, ^∗∗^*p* < 0.01, ^∗∗∗^*p* < 0.005, ^∗∗∗∗^*p* < 0.0001 vs. h-PLT (unpaired *t*-test and Newman–Keuls multiple-range test).

We tested the hypothesis that the expression of constitutive COX-1 was higher in diabetic-PLTs. Indeed, as shown in **Figure 5A**, higher levels of COX-1 were present in diabetic-PLTs (*p* < 0.0001).

We argued that phospho-AKT (pAKT) and phospho-ERK-2 levels may be enhanced by the pro-aggregating agents more in diabetic-PLTs than in healthy-PLTs. As shown in **Figure 5B**, in healthy-PLTs and diabetic-PLTs, we found similar protein expression of AKT and ERK-2 (ERK-1 was not investigated) (Woulfe, 2010). To evaluate the agonist-induced activation of the PI-3K and ERK pathways, washed platelet samples were stimulated by either collagen or AA and pAKT and pERK level measured. As expected, the amount of pAKT increased in response to either collagen (*p* < 0.0001 vs. baseline, for both groups) or AA (*p* < 0.0001 vs. baseline for both groups). However, the increase was significantly greater in diabetic-PLTs than healthy-PLTs, for both collagen (*p* < 0.01) and AA (*p* < 0.0001). Similarly, the amount of pERK-2 increased in response to either collagen (*p* < 0.0001 vs. baseline for both groups) or AA (*p* < 0.0001 vs. baseline for both groups). Yet, the increase was greater in diabetic-PLTs than healthy-PLTs, for both collagen (*p* < 0.05) and AA (*p* < 0.005).

### Isolated Hearts

Either healthy-PLTs or diabetic-PLTs were tested in isolated rat heart to verify their protective properties. Moreover, to ascertain the cardioprotective pathways activated by platelets, specific antagonists were used.

Of note, platelet infusion, as well as co-infusion with antagonists did not affect coronary perfusion pressure and left ventricular pressure (data not shown). These observations rule out impaired perfusion and plugged capillaries by platelets.

#### Infarct Size Was Reduced by Healthy-PLTs Pretreatment Only, via S1P Receptors and ERK/PI3K/PKC Pathways

**FIGURE 6:**
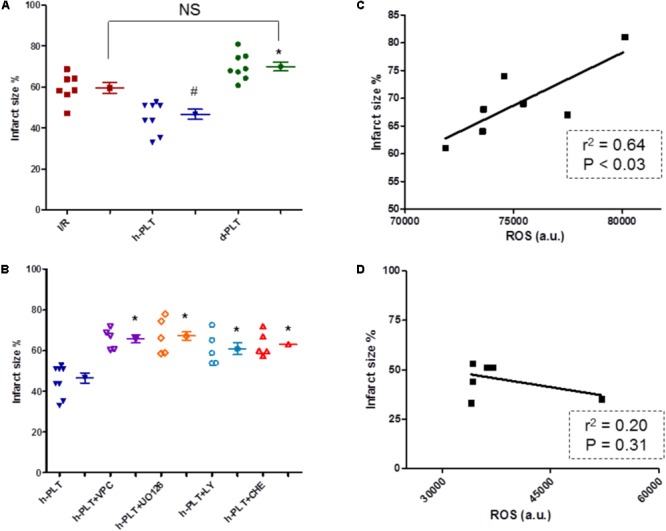
Infarct size expressed as percent of area at risk **(A,B)**, and platelet ROS-infarct size correlations **(C,D)**. **(A)** Washed platelets derived from healthy subjects (h-PLT) limited infarct size. The infarct size in hearts treated with platelets derived from diabetic patients (d-PLT) was higher than that observed in hearts pretreated with h-PLT. **(B)** The infarct size limitation effects of h-PLTs was abrogated by the blocker of S1P receptors, VPC23019, the antagonist of ERK1/2, U0126, the inhibitor of PI3K, LY or the antagonist of PKC, CHE. ^∗^*p* < 0.005 vs. h-PLT; ^#^*p* < 0.005 vs. I/R; NS, not significant (Newman–Keuls multiple-range test). **(C)** Correlation between ROS platelet levels and infarct size for the d-PLT group (*n* = 7; linear regression). **(D)** correlation between ROS platelet levels and infarct size for the h-PLT group (*n* = 7; linear regression).

After I/R, in control group infarct size was 59 ± 3% of the left ventricular mass. The 10 min pre-treatment with healthy-PLTs significantly (*p* < 0.005) reduced infarct size to 47 ± 2% of ventricular mass (**Figure 6A**). However, the 10 min pre-treatment with diabetic-PLTs did not reduce infarct size, which was 70 ± 2% of the left ventricular mass (Not Significant vs. I/R group; *p* < 0.005 vs. healthy-PLT group).

To study the role of myocardial S1P receptors and RISK pathway, healthy-PLTs were alternatively co-infused with one of the following compounds: VPC23019 to block cardiac S1P Receptors (types 1 and 3), U0126 to inhibit ERK1/2, LY294002 to inhibit PI3K, or Chelerythrine to block PKC. At the end of reperfusion, in these four inhibitor groups, infarct sizes (66 ± 2%, 61 ± 2%, 61 ± 3%, and 63 ± 3% of the ventricular mass, respectively) were similar to those observed in control I/R hearts, but significantly (*p* < 0.005 for all) higher than in healthy-PLTs group (**Figure 6B**).

The infusion of antagonists alone (VPC23019, U0126, LY294002, or Chelerythrine) did not affect infarct size, which was similar to control group (63 ± 2%, 59 ± 2%, 70 ± 5%, and 65 ± 3%, respectively, data not reported in the figure). These data are in agreement with previous studies (Penna et al., 2005; Vessey et al., 2009; Mochizuki et al., 2013).

#### Diabetic Platelet ROS Levels Correlate With Infarct Size

We hypothesized a correlation between platelet ROS levels (an index of platelet dysfunction) and infarct size. Indeed **Figure 6C** displays a good correlation between platelet ROS levels and infarct size for data of diabetic-PLTs, whereas between ROS levels in healthy-PLTs and infarct size there is a poor, not significant, correlation (**Figure 6D**).

#### Myocardial Phospho-ERK Is Involved in Healthy-PLT Induced Cardioprotection

**FIGURE 7:**
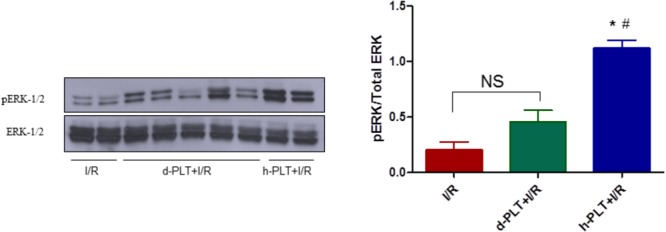
Phosphorylation of extracellular signal-regulated kinases (ERK-1/2) in lysates from hearts subjected to I/R only (control) or pre-treated with d-PLTs (washed platelets derived from diabetic patients) or h-PLTs (washed platelets derived from healthy subjects). Representative western blot of ERK-1/2 phosphorylation and total ERK-1/2 of protein extract from control I/R (*n* = 4), d-PLTs (*n* = 5), and h-PLTs (*n* = 8) hearts. Histograms represent the ratio of phosphorylated over total ERK protein expression. Data suggest that pretreatment with h-PLTs induces an increase in ERK-1/2 phosphorylation with respect to I/R only. ^∗^*P* < 0.0001 vs. I/R; ^#^*P*< 0.001 vs. d-PLTs (Newman–Keuls multiple-range test).

To corroborate data obtained with inhibitors of survival kinases, we measured ERK-1/2 expression and phosphorylation in myocardial tissue of hearts treated with healthy-PLTs. The phosphorylation of ERK-1/2 was greater in hearts pre-treated with healthy-PLTs compared to hearts subjected to I/R (*p* < 0.0001; **Figure 7**). A three–fourfold higher pERK/total ERK ratio was observed in healthy-PLT pretreated hearts compared to I/R hearts. In diabetic-PLT pretreated hearts the ratio was not different from that in I/R hearts.

## Discussion

The main novel findings of this study are: (1) the heart pretreatment with platelets of healthy humans (healthy-PLTs) reduces infarct size, (2) the cardioprotective properties of healthy-PLTs depend on myocardial S1P receptor and RISK (PI3K-ERK-1/2-PKC) pathway activation, (3) the cardioprotective properties are lost by platelets from T2DM patients (diabetic-PLTs), which release less S1P than healthy-PLTs after mechanical and chemical stimulation, *in vitro*, and (4) the higher is the ROS level in PLTs the higher is infarct size in hearts pretreated with diabetic PLTs.

### Pretreatment With Healthy Human PLTs Reduces Infarct Size via S1P Receptor Activation

Pre-treatment with healthy human PLTs limits infarct size in the Langendorff isolated rat heart. Thus, platelets may interact with uninjured organ to trigger protection in hearts subsequently subjected to I/R *ex vivo*. Data are in agreement with previous studies showing that perfusion of isolated rat hearts with platelets from normal rats protects against myocardial dysfunction caused by I/R, improving both biochemical and dynamic cardiac parameters (Yang et al., 1993, 1994; Yang and Mehta, 1994; Mehta et al., 1999). Similar cardioprotection was observed in hearts perfused with platelet supernatant (Yang et al., 1998, 1999), thus suggesting an important role for factors released by platelets.

In our model, the treatment of hearts with VPC23019, an S1PR_1_ and S1PR_3_ antagonist (Davis et al., 2005), abrogates cardioprotection induced by healthy-PLTs. Therefore, it is likely that healthy human platelets protect the hearts against I/R injury *via* an S1P mediated mechanism. Indeed, S1P is a platelet factor whose release originates from two pools: one constitutively secreted and located at the plasma membrane, and the other rapidly phosphorylated upon stimulation and mobilized from granules (Yatomi et al., 1995; Tani et al., 2005). This release in the supernatant is influenced by mechanical (stirring) and chemical (collagen) stimuli *in vitro*.

Intriguingly, S1P is a crucial signal molecule abundantly stored in platelets, which displayed a variety of cellular functions, including cardioprotective properties (Chalfant and Spiegel, 2005; Cohen et al., 2016). S1P is stored within the platelets and is released in certain circumstances (Tani et al., 2005). Several studies performed in animal and *in vitro* models have proposed that S1P possesses cardioprotective effects (Kupperman et al., 2000; Jin et al., 2002; Zhang et al., 2007; Karliner, 2013; Maceyka and Spiegel, 2014). Moreover, S1P protects cultured rat neonatal cardiomyocytes from ischemia-induced cell death (Jin et al., 2002; Karliner, 2013). Since only S1PR_1_, S1PR_2_, and S1PR_3_ are the receptor subtypes expressed in cardiac myocytes and endothelial cells (Peters and Alewijnse, 2007; Karliner, 2009; Means and Brown, 2009; Zhang et al., 2013), it is likely that these receptors play a major role in cardioprotection by healthy-PLT pretreatment. Indeed, in the heart binding of S1P to S1PR_1_ leads to activation of ERK1/2 and binding to the S1PR_3_ receptor promotes the activation of PI3K/AKT (Knapp, 2011; Somers et al., 2012). Nevertheless, it is likely that the cardioprotective effect of S1P is the result of a co-operation of the three receptors expressed by the heart (Means et al., 2007; Vessey et al., 2009; Cohen et al., 2016). That is why blocking both S1PR_1_ and S1PR_3_ with VPC23019, we blocked the platelet cardioprotective effects. It is unlikely that VPC23019 interfered with platelet S1P receptors because platelets express mainly S1PR_2_ and S1PR_4_ (Randriamboavonjy et al., 2009; Onuma et al., 2017) (see also below “Methodological considerations *and limitations of the study*”).

### Infarct Size Limitation by Healthy-PLTs Is Reversed by RISK (PI3K, ERK-1/2, and PKC) Pathway Inhibition

**FIGURE 8:**
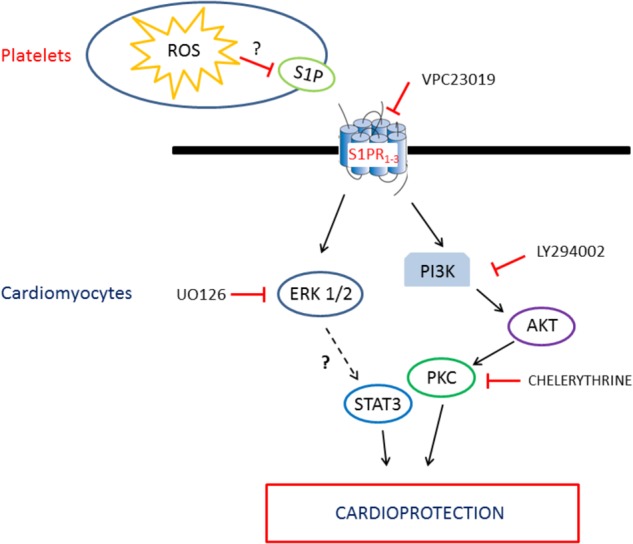
Schematic representation of the intracellular pathway involved in the platelet/S1P cardioprotection.

It has been reported that nitric oxide release may mediate cardioprotection by S1P (Egom et al., 2011). Since in the RISK pathway AKT is upstream to nitric oxide synthase, we verified whether inhibitors of RISK pathway may avoid platelet-induced cardioprotection. Indeed, healthy-PLT-induced cardioprotection was completely abolished by pre-treating the heart with LY294002 to block PI3K, or Chelerythrine to block PKC, as well as U0126 to inhibit ERK-1/2. Thus, suggesting a pivotal role of these survival kinases in the platelet-induced cardioprotection (**Figure 8**). A role which is corroborated by myocardial Western blot data, revealing elevated levels of *pERK-1/2* in healthy-PLT pretreated hearts only. This kinase phosphorylation is usually observed in conditioning treatments, with a peak of the pERK**/**total ERK ratio 10–15 min after the end of ischemia and a progressive reduction thereafter (Ferdinandy et al., 2014; Penna et al., 2014; Cohen and Downey, 2015). A three–fourfold higher pERK/total ERK ratios in hearts pretreated with healthy-PLTs is a strong indication of kinase involvement in protection, especially if we consider that healthy-PLT pretreated hearts have just a 30% more vital tissue than control I/R hearts.

Moreover, since STAT3 seems to be downstream to ERK (Knapp, 2011; Somers et al., 2012) in ongoing experiments we tested whether healthy or diabetic PLTs can activate myocardial STAT3. Indeed, we found that h-PLT treated hearts display a higher pSTAT3 level than d-PLT treated hearts (see **Supplementary Materials**).

However, it has been also shown that in some circumstances, platelets may contribute to initiation and propagation of I/R myocardial injury (Aiken et al., 1981; Vanhoutte and Houston, 1985; Yee et al., 1986; Patel et al., 2001; Seligmann et al., 2013). These apparently conflicting reports may reflect different experimental designs: platelets introduced into the coronary system of heart preparations not before but during or after ischemia, atherosclerotic coronary arteries or coronary endothelial barrier failure. In particular, it has been suggested that the deleterious effects of platelets on reperfused myocardium depend on their activation status, which is enhanced by the previous exposure to I/R injured myocardium (Mirabet et al., 2002). In other words, it seems that in I/R scenario platelet deleterious effects are due to interaction with injured tissues and/or platelet pre-activation. Therefore, we studied the “*hyperreactive*” diabetic-PLTs.

### Cardioprotective Properties Are Lost by Diabetic-PLTs

The cardioprotective properties seen for healthy-PLTs are lost by diabetic-PLTs. In fact, infarct size after intracoronary pretreatment with diabetic-PLTs is significantly higher than that observed in hearts pre-treated with healthy-PLTs and not significantly different from the control I/R hearts. This is in agreement with the idea that activated platelets lose the cardioprotective properties (Mirabet et al., 2002).

Multiple factors may cause dysregulation of platelet signaling pathways leading to a hyper-reactive platelet phenotype. Indeed, diabetic subjects of our study were not only characterized by hyperglycemia and uncontrolled diabetes but also by other dysmetabolic conditions such as obesity and dyslipidemia, commonly associated with T2DM, and each of them may contribute to the diabetic-PLT hyper-reactivity observed in this population (Russo, 2012; Morange and Alessi, 2013). In this study, we confirm that, in comparison with healthy-PLTs, platelets from T2DM patients show: (a) higher aggregating response to agonists (b) higher basal and AA-induced production of ROS, (c) enhanced levels of COX-1 and (d) increased levels of *phospho-AKT* and *phospho-ERK-2* upon stimulation with agonists, more in diabetic than healthy platelets, thus confirming the hyper-reactivity of diabetic platelets. However, unlike myocardium, these two kinases are not linked to a survival signaling in platelets (Kovacsovics et al., 1995; Aharonovitz and Granot, 1996). Indeed, *following stimuli*, ERK and AKT regulate platelet activation and function, such as adhesion or secretory changes, and are actively involved in conveying signals from several pathways leading to platelet activation (Kovacsovics et al., 1995; Aharonovitz and Granot, 1996).

Our findings on platelet function in T2DM subjects fit into the general picture of the current research that describes platelet hyper-reactivity as responsible, at least in part, of the prothrombotic state in T2DM (Jung et al., 2015). We also demonstrate that higher ROS levels in diabetic-PLTs are, at least in part, due to enhanced production by NADPH-oxidase and COX-1 activity. Indeed, ROS generation in diabetic-PLTs is mostly produced by COX-1 activity, as shown by the significant reduction of ROS levels using the COX-1 inhibitor, INDO. A role is also played by the pro-oxidant enzyme NADPH-oxidase, as shown by the significant reduction of ROS levels when diabetic-PLTs were preincubated with two different NADPH-oxidase inhibitors, APO or DPI. Several reports have suggested that ROS represent a new modulator of platelet activity and ROS generated by platelets have a direct role in the control of their responsiveness (Vazzana et al., 2012).

The correlation between platelet ROS levels in diabetic platelets and the extension of infarct size suggests that the redox status in these platelets may play a pivotal role in influencing their cardiac effects. The absence of correlation for healthy platelets suggests that a certain threshold of ROS should be reached to affect protective properties. Of note, a complete prevention of the post-ischemic cardio-depressive effects by platelets – administrated during ischemia or reperfusion on isolated guinea pig hearts – was observed after platelet pretreatment with the NADPH-oxidase inhibitor, DPI (Seligmann et al., 2013). These data are in agreement with our data that ROS levels in hyperactive platelets are correlated to the extension of I/R injury and that platelet ROS derive, at least in part, from NADPH-oxidase. Nevertheless, correlation is not causality and our results need further studies to clarify the role of ROS.

Here, we show that diabetic platelets release less S1P when mechanically (*stirring*) or chemically (collagen) stimulated. Our findings are in line with the observation that diabetic platelets have a dysregulation of the S1P component ready to be mobilized (Książek et al., 2015). Moreover, the fact that S1P release is compromised is in agreement with the observation that diabetic platelets display higher expression of signs and factors of dysregulation, including higher ROS amount produced at basal condition and after stimulation with AA. In other words, the loss of protective properties observed in diabetic platelets could be linked to the altered redox and aggregatory conditions of these platelets that would alter the modality and/or quantity of released S1P. However, this hypothesis needs more experiments to be fully ascertained. Also, the consequences and mechanisms of S1P dysregulation in diabetic platelets need to be further investigated.

### Methodological Considerations and Limitations of the Study

The main aim of the present study was to ascertain the mechanisms of platelet-induced cardioprotection. However, diabetic PLTs were not protective and it remains to ascertain the reasons for this ineffectiveness. Indeed, the use of a normal heart allowed us to rule out that the lack of protection is due to myocardial resistance to protection, typical of diabetes and other co-morbidities (Ferdinandy et al., 2014). The defect is likely within diabetic platelets: the impaired release of S1P may prevent protection from diabetic-PLTs. However, it cannot be so easily determined because these platelets are ineffective in influencing the infarct size in Langendorff hearts: blocking myocardial S1P receptors or RISK pathway will not reveal the mechanism of diabetic-PLTs ineffectiveness. It may be also argued that blocking ROS production with antioxidants in washed platelets may reverse the platelet defect but ROS blockers/scavengers may interfere directly with myocardial I/R injury and/or platelet S1P release. Therefore, future studies should envision appropriate protocols to solve these open questions and, in particular, the reasons for diabetic-PLTs ineffectiveness.

Although the Langendorff’s model, like all experimental paradigms, has disadvantages and advantages, it is a useful paradigm for studying the role of cardioprotective procedures (Bell et al., 2011). Indeed, Langendorff’s model allows to subjecting the heart to a “clean” test, and to remove unwanted interferences by blood cells, protein and other “confounding variables,” such as temperature, perfusion, and neuro-hormonal influences. Indeed, experiments performed on Langendorff’s murine model provided very strong data on the cardioprotective effect of S1P (Jin et al., 2002; Lecour et al., 2002; Vessey et al., 2006, 2008).

It is unlikely that the S1PR_1/3_ antagonist, VPC23019, interfered with the capacity of platelets to release S1P. It has been reported that human platelets may express types 1, 2, and 4 of S1PRs (Hla et al., 2012; Urtz et al., 2015). However, Fleming and co-workers were able to detect S1PR_2_, but not S1PR_1_ on human platelets (Randriamboavonjy et al., 2009); in this study, S1PR_2_ displayed a pro-aggregating role. In another study, it has been suggested that S1P suppresses collagen-induced aggregation *via* S1PR_4_, but not through S1PR_1_ in human platelets (Onuma et al., 2017). Therefore, it seems that human platelets express S1PR_2_ and S1PR_4_, with a pro- and anti-aggregant role, respectively. Whether S1PR_1_ is present and/or play a role seems controversial. Therefore, VPC23019 may not interfere with the principal receptors present on platelets, but it is specific for the receptors expressed by the cardiac tissues. Nevertheless, S1PRs are involved in platelet-induced cardioprotection.

We used a commercially available ELISA kit for the assessment of S1P release in washed platelets. This differs from protocol usually performed to measure S1P levels in serum or plasma samples where high-density lipoproteins are the major plasma carriers for S1P. Clearly, S1P values in our study are not comparable to those obtained in blood/plasma samples.

We used washed platelets, therefore it is unlikely that the S1P was already in the perfusate. Only the S1P released by circulating platelets may be involved in the cardioprotective effect. Indeed, stirring may simulate the circulating conditions that allow the release of larger quantities of S1P. Nevertheless, in future studies, it might be worthwhile to test whether platelet-derived supernatant can be protective *via* S1P mechanism.

*In summary*, three key observations regarding the platelet function and role in I/R scenario were made in this study: (1) S1P/S1PRs play important roles in determining healthy-platelet-induced cardioprotection *via* myocardial RISK pathway, in an isolated rat heart model, (2) these cardioprotective properties are lost by diabetic platelets, which produce higher levels of ROS and lower levels of S1P when stimulated *in vitro*, and (3) infarct size correlates with the amount of ROS produced by diabetic platelets.

## Conclusion

Here we provide evidence that human healthy platelets exert cardioprotective effects *via* activation of an S1P related mechanism. It is likely that myocardial RISK pathway plays a pivotal role in platelet-induced cardioprotection. Our data also suggest that alterations in ROS production and in S1P release may be involved in the impairment of function and the loss of cardioprotective properties by diabetic platelets. Since plasma S1P concentration is reduced in patients with myocardial infarction (Knapp et al., 2009), its modulation by drugs targeting platelets might be very important and need to be better understood. Since exosomes greatly contribute to platelet activity, whether and how the platelet-induced cardioprotection is linked to the well-known exosome-induced cardioprotection (Barile et al., 2014, 2018) remain to be elucidated. Further studies in this direction are warranted.

## Author Contributions

CP, IR, and PP conception and design of the experiments. CB and IR performed the experiments on isolated platelets and western blot analyses for both platelets and myocardium. CP, FT, and SF performed the I/R experiments on isolated hearts. CB, CP, FC, FT, PP, IR, and SF analysis and interpretation of the data. CP, IR, SF, and PP drafted the article. CP, FC, PP, IR, SF, and SG revised the manuscript critically for important intellectual content.

## Conflict of Interest Statement

The authors declare that the research was conducted in the absence of any commercial or financial relationships that could be construed as a potential conflict of interest.

## Supplementary Material

FIGURE S1 Expression of extracellular signal-regulated kinases (ERK-1/2) in lysates from hearts subjected to ischemia/reperfusion only (I/R) or pre-treated with platelets from healthy subjects (hPLT+IR).

FIGURE S2 Phosphorylation of extracellular signal-regulated kinases (pERK-1/2) in lysates from hearts subjected to ischemia/reperfusion only (I/R) or pre-treated with platelets from healthy subjects (hPLT+IR).

FIGURE S3 Expression of protein kinase B (AKT) in platelets from healthy and Type 2 Diabetes Mellitus (T2DM) subjects in the absence (basal) or in the presence of collagen (coll) or arachidonic acid (AA).

FIGURE S4 Expression of ciclooxygenase-1 (COX-1) in platelets from healthy and Type 2 Diabetes Mellitus (T2DM) subjects.

FIGURE S5 Expression of extracellular signal-regulated kinase-2 (ERK-2) in platelets from healthy and Type 2 Diabetes Mellitus (T2DM) subjects in the absence (basal) or in the presence of collagen (coll) or arachidonic acid (AA).

FIGURE S6 Phosphorylation of protein kinase B (pAKT) in platelets from healthy and Type 2 Diabetes Mellitus (T2DM) subjects in the absence (basal) or in the presence of collagen (coll) or arachidonic acid (AA).

FIGURE S7 Phosphorylation of extracellular signal-regulated kinases (pERK-1/2) in platelets from healthy and Type 2 Diabetes Mellitus (T2DM) subjects in the absence (basal) or in the presence of collagen (coll) or arachidonic acid (AA).

FIGURE S8 Expression of vinculin in platelets from healthy and Type 2 Diabetes Mellitus (T2DM) subjects.

FIGURE S9 Expression of extracellular signal-regulated kinases (ERK-1/2) in lysates from hearts subjected to ischemia/reperfusion only (I/R), pre-treated with platelets from Type 2 Diabetes Mellitus (T2DM) subjects (dPLT+IR) or healthy (hPLT+IR).

FIGURE S10 Phosphorylation of extracellular signal-regulated kinases (pERK-1/2) in lysates from hearts subjected to ischemia/reperfusion only (I/R), pre-treated with platelets from Type 2 Diabetes Mellitus (T2DM) subjects (dPLT+IR) or healthy (hPLT+IR).

FIGURE S11 Phosphorylation of STAT-3 (pSTAT-3) in lysates from hearts subjected to ischemia/reperfusion only (I/R), pre-treated with platelets from healthy (hPLT+IR) or Type 2 Diabetes Mellitus (T2DM) subjects (dPLT+IR).
