# Supplementary figures and images for: Cardioprotective Properties of Human Platelets Are Lost in Uncontrolled Diabetes Mellitus: A Study in Isolated Rat Hearts
Source: Front Physiol. 2018 Jul 10;9:875. doi: 10.3389/fphys.2018.00875 (PMC6048273; doi:10.3389/fphys.2018.00875)

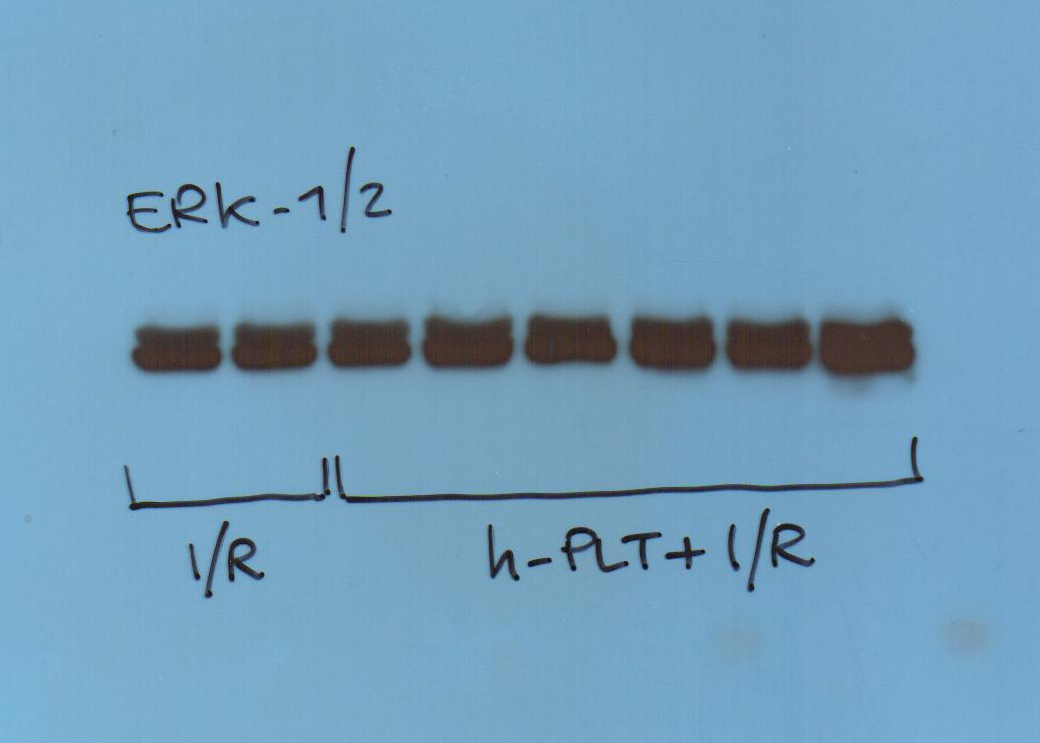

Supplement: FIGURE S1 — Expression of extracellular signal-regulated kinases (ERK-1/2) in lysates from hearts subjected to ischemia/reperfusion only (I/R) or pre-treated with platelets from healthy subjects (hPLT+IR). [file Image_1.JPEG]

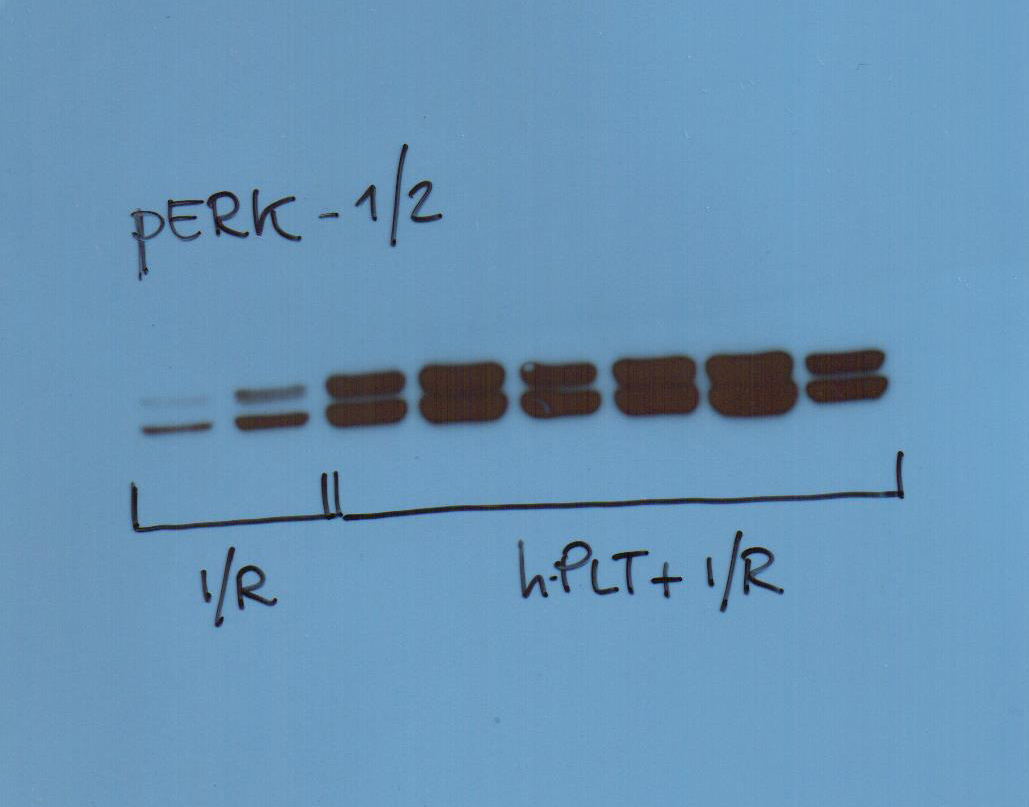

Supplement: FIGURE S2 — Phosphorylation of extracellular signal-regulated kinases (pERK-1/2) in lysates from hearts subjected to ischemia/reperfusion only (I/R) or pre-treated with platelets from healthy subjects (hPLT+IR). [file Image_2.JPEG]

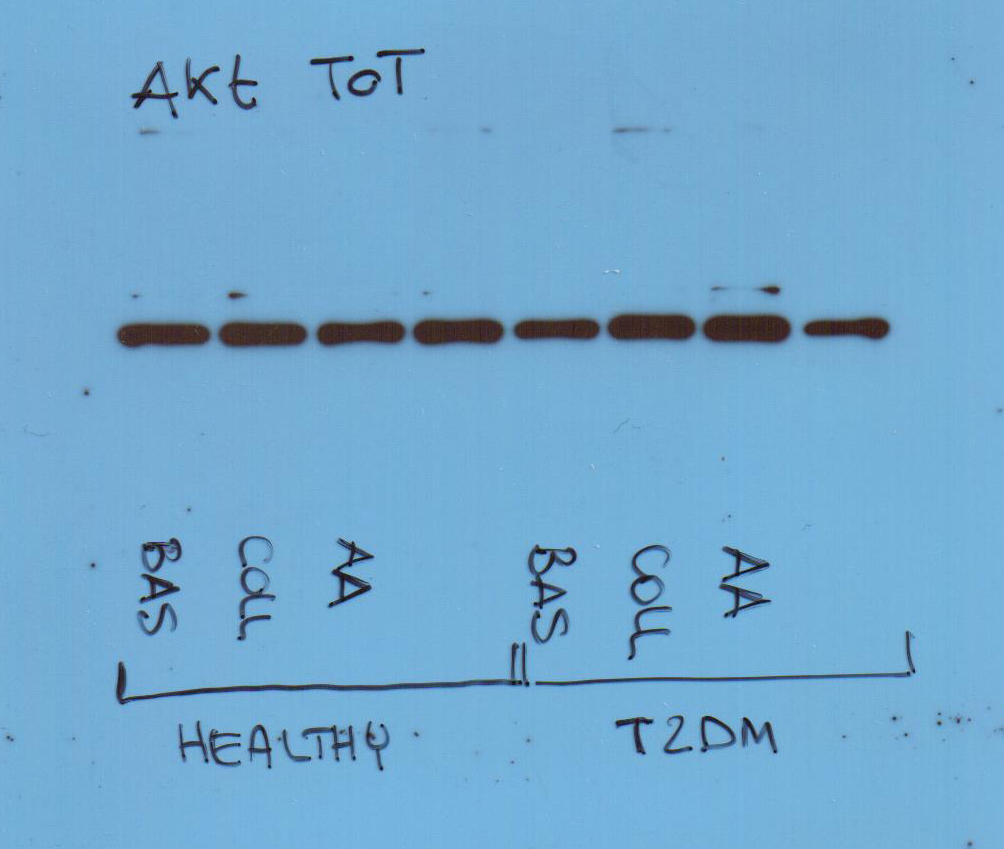

Supplement: FIGURE S3 — Expression of protein kinase B (AKT) in platelets from healthy and Type 2 Diabetes Mellitus (T2DM) subjects in the absence (basal) or in the presence of collagen (coll) or arachidonic acid (AA). [file Image_3.JPEG]

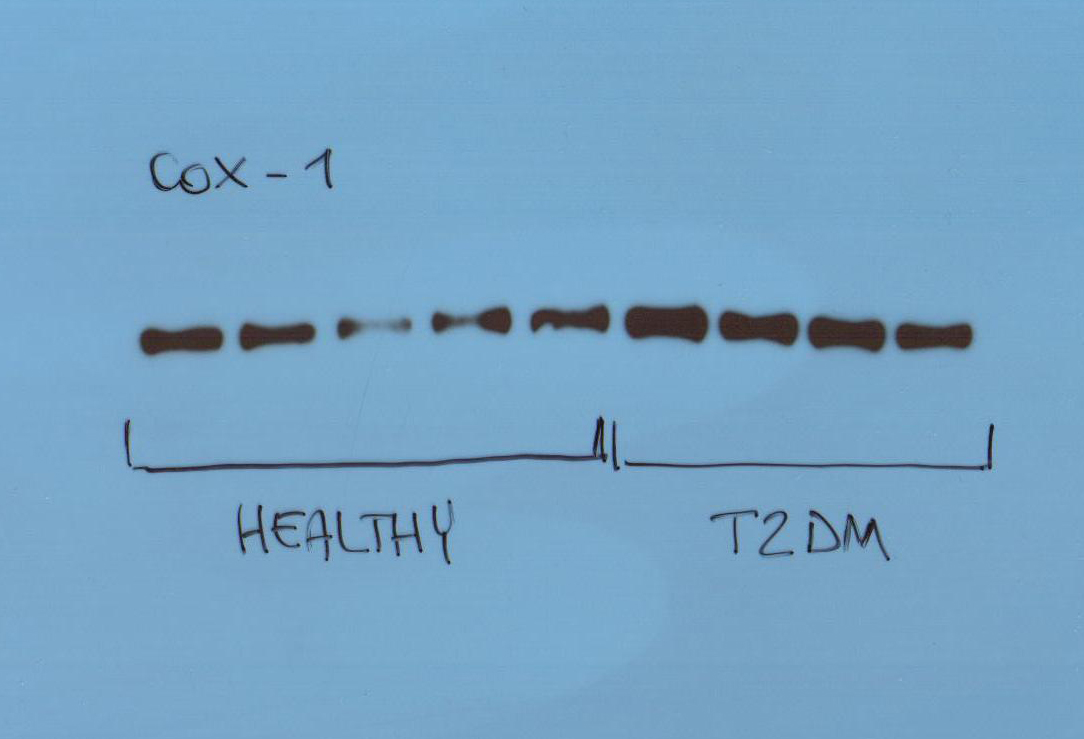

Supplement: FIGURE S4 — Expression of ciclooxygenase-1 (COX-1) in platelets from healthy and Type 2 Diabetes Mellitus (T2DM) subjects. [file Image_4.JPEG]

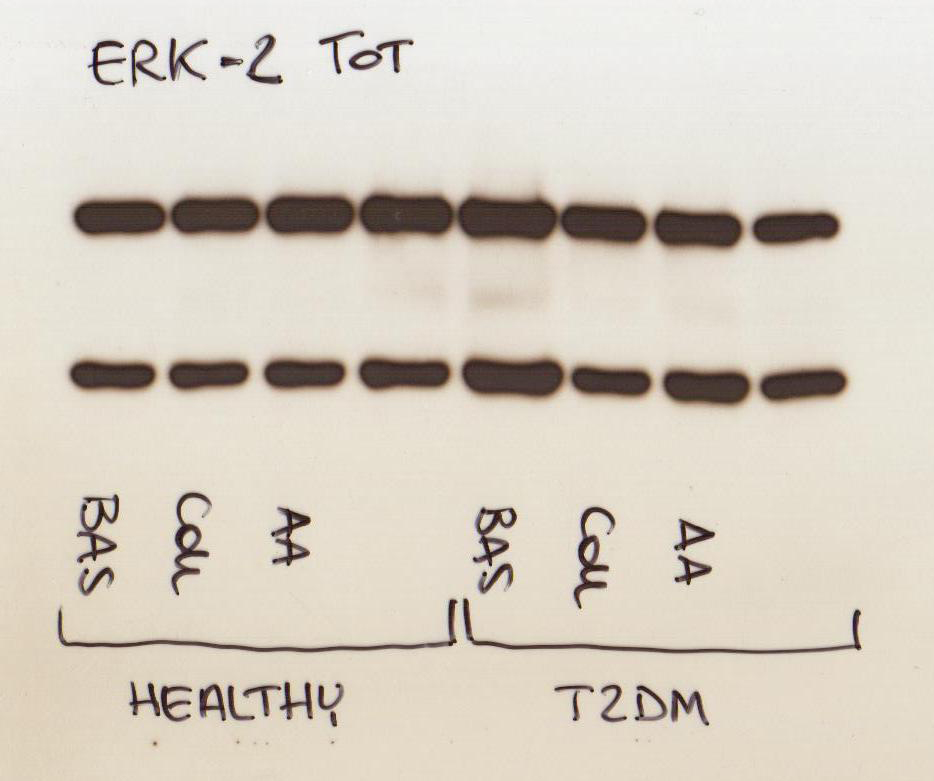

Supplement: FIGURE S5 — Expression of extracellular signal-regulated kinase-2 (ERK-2) in platelets from healthy and Type 2 Diabetes Mellitus (T2DM) subjects in the absence (basal) or in the presence of collagen (coll) or arachidonic acid (AA). [file Image_5.JPEG]

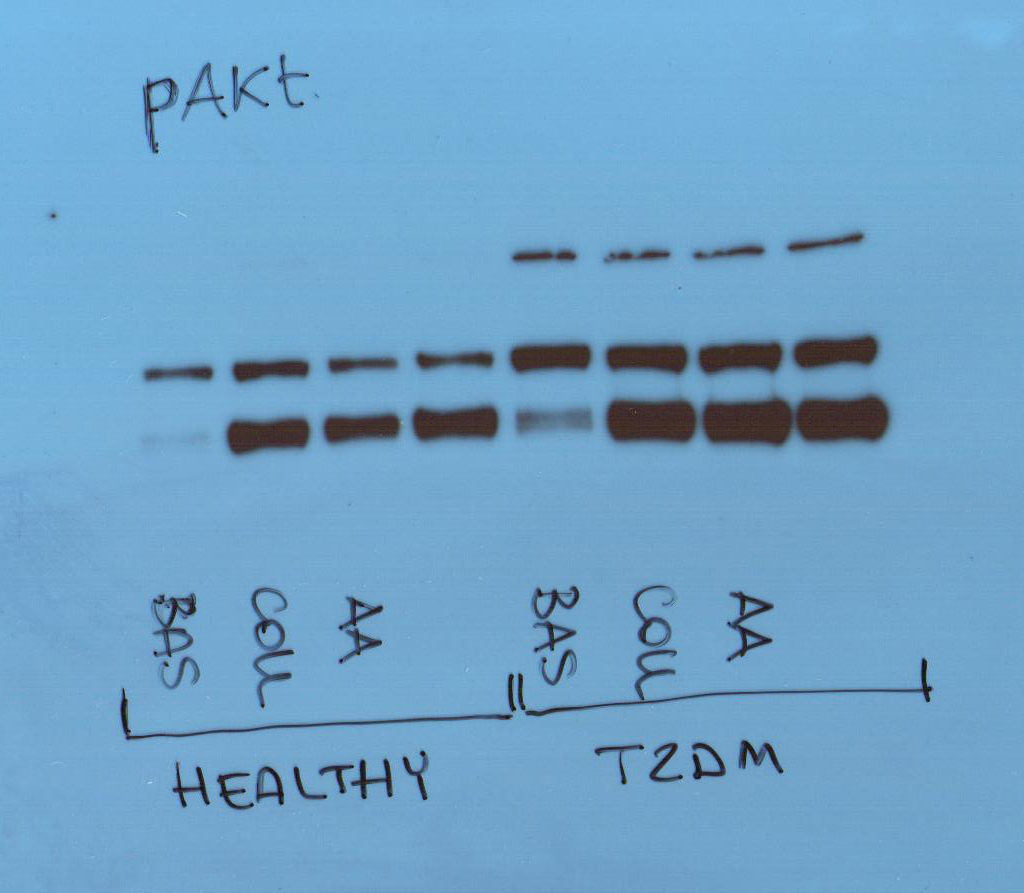

Supplement: FIGURE S6 — Phosphorylation of protein kinase B (pAKT) in platelets from healthy and Type 2 Diabetes Mellitus (T2DM) subjects in the absence (basal) or in the presence of collagen (coll) or arachidonic acid (AA). [file Image_6.JPEG]

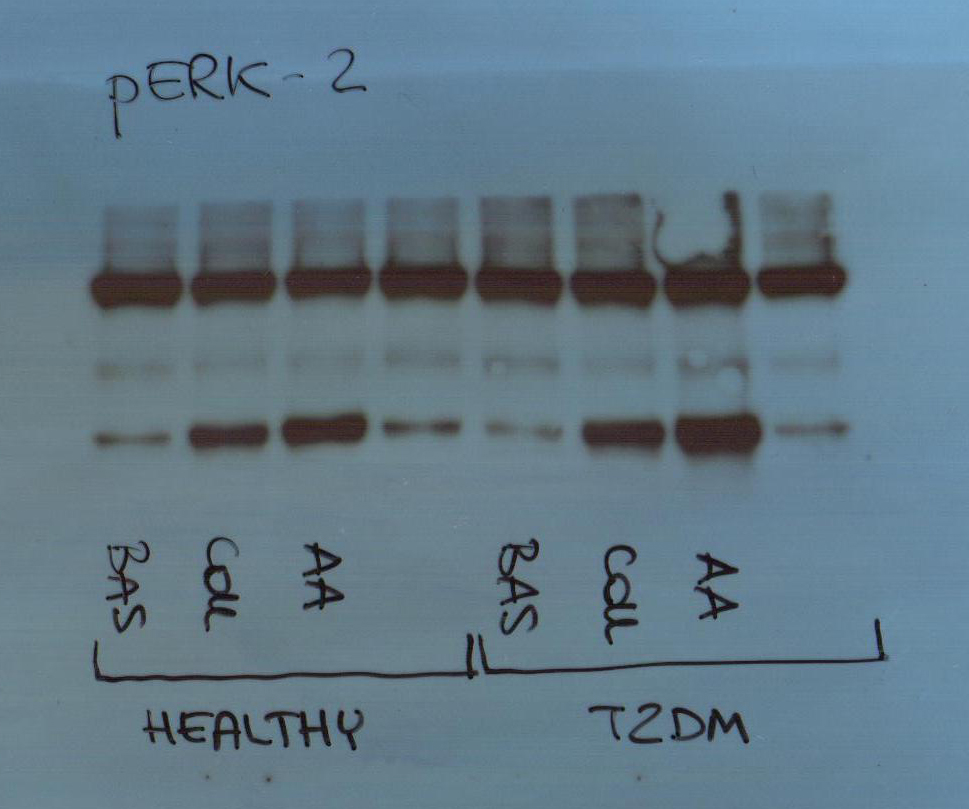

Supplement: FIGURE S7 — Phosphorylation of extracellular signal-regulated kinases (pERK-1/2) in platelets from healthy and Type 2 Diabetes Mellitus (T2DM) subjects in the absence (basal) or in the presence of collagen (coll) or arachidonic acid (AA). [file Image_7.JPEG]

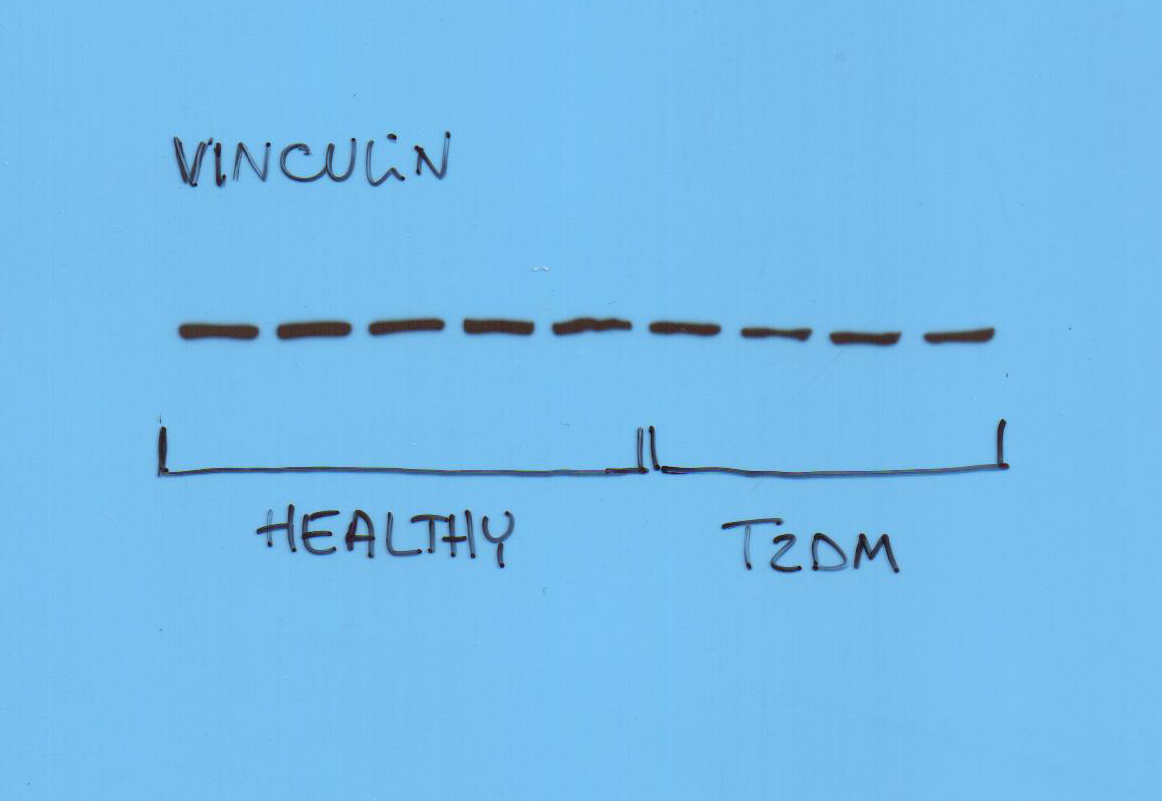

Supplement: FIGURE S8 — Expression of vinculin in platelets from healthy and Type 2 Diabetes Mellitus (T2DM) subjects. [file Image_8.JPEG]

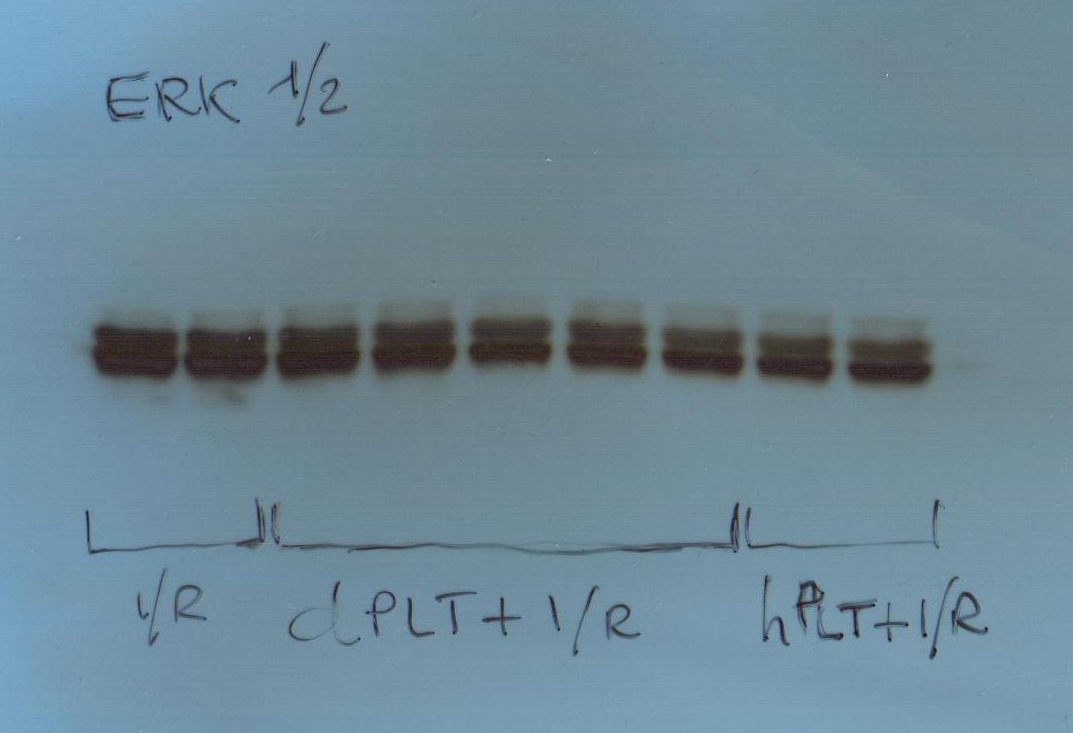

Supplement: FIGURE S9 — Expression of extracellular signal-regulated kinases (ERK-1/2) in lysates from hearts subjected to ischemia/reperfusion only (I/R), pre-treated with platelets from Type 2 Diabetes Mellitus (T2DM) subjects (dPLT+IR) or healthy (hPLT+IR). [file Image_9.JPEG]

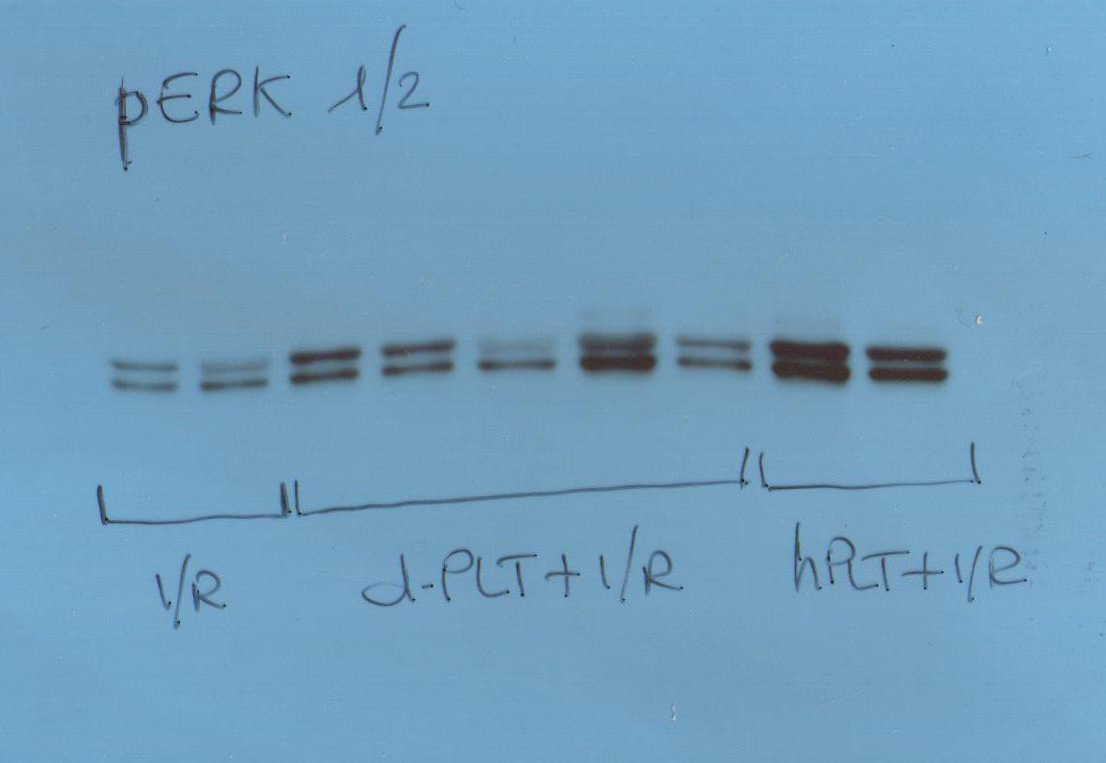

Supplement: FIGURE S10 — Phosphorylation of extracellular signal-regulated kinases (pERK-1/2) in lysates from hearts subjected to ischemia/reperfusion only (I/R), pre-treated with platelets from Type 2 Diabetes Mellitus (T2DM) subjects (dPLT+IR) or healthy (hPLT+IR). [file Image_10.JPEG]

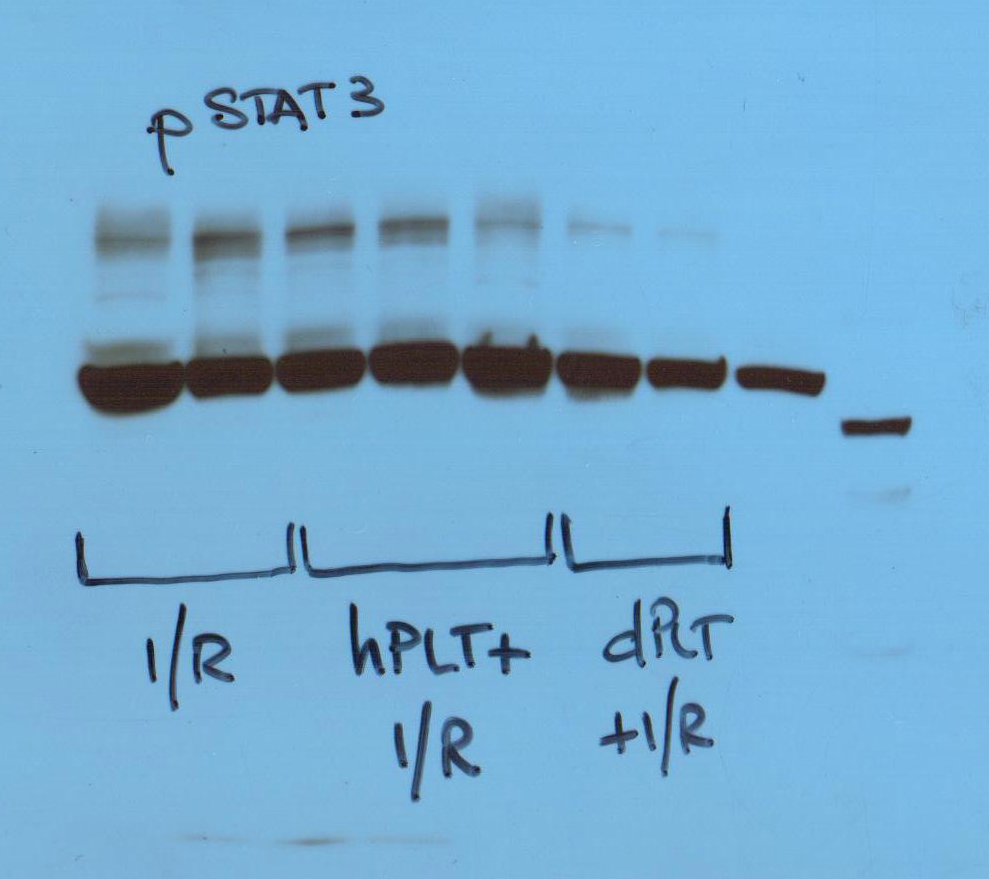

Supplement: FIGURE S11 — Phosphorylation of STAT-3 (pSTAT-3) in lysates from hearts subjected to ischemia/reperfusion only (I/R), pre-treated with platelets from healthy (hPLT+IR) or Type 2 Diabetes Mellitus (T2DM) subjects (dPLT+IR). [file Image_11.JPEG]
